# Metaplasia in the Stomach—Precursor of Gastric Cancer?

**DOI:** 10.3390/ijms18102063

**Published:** 2017-09-27

**Authors:** Hiroto Kinoshita, Yoku Hayakawa, Kazuhiko Koike

**Affiliations:** Graduate School of Medicine, Department of Gastroenterology, The University of Tokyo, 7-3-1, Hongo, Bunkyo-ku, Tokyo 113-8655, Japan; kinoshita-tky@umin.ac.jp (H.K.); kkoike-tky@umin.ac.jp (K.K.)

**Keywords:** gastric cancer, intestinal metaplasia, Spasmolytic polypeptide expressing metaplasia (SPEM), *Helicobacter pylori*, stem cells, lineage tracing

## Abstract

Despite a significant decrease in the incidence of gastric cancer in Western countries over the past century, gastric cancer is still one of the leading causes of cancer-related deaths worldwide. Most human gastric cancers develop after long-term *Helicobacter pylori* infection via the Correa pathway: the progression is from gastritis, atrophy, intestinal metaplasia, dysplasia, to cancer. However, it remains unclear whether metaplasia is a direct precursor of gastric cancer or merely a marker of high cancer risk. Here, we review human studies on the relationship between metaplasia and cancer in the stomach, data from mouse models of metaplasia regarding the mechanism of metaplasia development, and the cellular responses induced by *H. pylori* infection.

## 1. Human Gastric Cancer Pathogenesis

Gastric cancer was the leading cause of cancer-related death in the United States and other Western countries until the 1930s–1940s. Despite a significant decrease in the incidence of gastric cancer in Western countries over the past century, gastric cancer is still one of the leading causes of cancer-related death worldwide [1]. Most human gastric cancers arise after long-term *Helicobacter pylori* infection via a multi-step histopathological cascade known as the Correa pathway, which involves the following steps: gastritis, atrophy, intestinal metaplasia (IM), dysplasia, and ultimately cancer [2,3,4]. Metaplasia is the replacement of one differentiated cell type with another mature differentiated cell type that is not normally present in a specific tissue [5]. IM in the stomach is histologically defined as changes in gland structure (to resemble intestinal glands) and the presence of intestine-specific lineage cells that are not present in the normal stomach such as goblet cells and Paneth cells. IM in the stomach is molecularly characterized by expression of intestine-specific transcription factors of the caudal type homeobox (Cdx) family, CDX1 and CDX2. 

During *H. pylori* infection, gastric atrophy, histologically defined as the loss of both chief and parietal cells, progresses from the antrum to the corpus along the lesser curvature, unless autoimmune gastritis is also present [6,7]. The histological extent of atrophy, as well as the endoscopic grade, is related to the risk of gastric cancer development [7,8,9,10,11,12,13]. As conventional endoscopy is not very accurate when used to diagnose IM (the sensitivity and specificity of endoscopic diagnosis of IM based on histology were about 25% and 90% [14]), histological analysis of multiple biopsy specimens is usually required to define the IM grade. Several studies have shown that IM is associated with a higher risk of cancer in the corpus than in the antrum alone (hazard ratios were 3.6 in the patients with IM in the antrum only and 3.8 for those with IM in the corpus), suggesting that IM progresses together with atrophy and predicts the gastric cancer risk [7,13,15]. Although *H. pylori* eradication to prevent cancer has been attempted in recent years [16,17], patients with high-level atrophy and IM are thought to be at especially high risk of gastric cancer even after *H. pylori* eradication [15,18]. Several recent studies have shown that IM may not improve after such eradication, suggesting that IM is irreversible [19,20], although other studies reported that eradication somewhat reduced the extent of IM [21,22,23]. Further prospective studies are needed to fully elucidate whether *H. pylori* eradication enables recovery from atrophy and IM, and eventually prevention for cancer development.

IM can be divided into two subtypes, complete and incomplete IM (Table 1). Complete IM resembles small intestinal glands with loss of gastric mucins (MUC1, MUC5AC, and MUC6) as well as the presence of eosinophilic enterocytes with brush borders, well-defined goblet cells, and occasional Paneth cells. In contrast, incomplete IM (also known as gastric or mixed type IM) resembles colonic glands in terms of structure, with multiple, irregular intracytoplasmic mucin droplets and absence of a brush border, and it often expresses gastric and intestinal mucins simultaneously [24]. Both types of IM express acid mucins (which contain substantial amounts of sialic acid and/or sulfate residues), which can be detected by Alcian blue staining at pH 2.5. In addition, incomplete IM expresses sulfomucins (which contain predominantly sulfate residues), which are stained brown by high iron diamine (HID), whereas complete IM expresses sialomucins (which contain predominantly sialic acid residues), which are not detected by HID staining [25]. One study found that incomplete IM was more proliferative than complete IM [26]. 

Although earlier researchers proposed that gastric cancer arises from “intestinalized” glands in the stomach, it remains unclear whether IM is a direct precursor of gastric cancer or merely a marker of high cancer risk. Given that most metaplastic cells are non-proliferative and post-mitotic, complete IM (in particular) likely represents a differentiated cell lineage distinct from dysplasia or cancer. In fact, complete IM is merely a weak risk factor for gastric cancer, and it may even suppress cancer development [27,28]. The expression of CDX2 is also associated with better outcome in gastric cancer patients [29]. In contrast, incomplete IM appears to be a more significant risk factor for gastric cancer [27,28,30,31]. The incidence of incomplete IM is correlated with the extent of total IM. Thus, incomplete IM is considered to be a more advanced stage of IM among the multiple steps of carcinogenesis. However, it remains to be elucidated whether complete IM progresses directly to incomplete IM, or whether both types of IM emerge independently. 

Several reports on the clonalities of human gastric specimens have revealed that both IM and dysplastic glands are clonal and can form large clonal patches via gland fission [32]. This process (termed “field cancerization”) suggests that both dysplasia and metaplasia in the stomach originate from gastric stem cells. Somatic mutations commonly seen in gastric cancer, such as tumor protein p53 (*TP53*) mutation, can be found in non-dysplastic IM, which may argue in favor of IM leading directly to dysplasia and cancer [33,34]. In fact, the aforementioned study revealed that the same somatic mutations of *TP53* or adenomatous polyposis coli (*APC*) were found in both IM and dysplasia in some of the cases [32]. The cellular kinetic changes evident in IM, in which the apoptotic index is significantly attenuated, (but the proliferation index is similar, to that of the non-IM epithelium), may also favor neoplasm formation [35]. However, it remains unclear whether IM glands can directly transform into dysplastic glands, or whether they simply share a common stem cell origin. In any case, it is important to unravel the molecular mechanisms of metaplasia development to develop effective approaches to cancer prevention and treatment.

## 2. Mouse Models of Metaplasia

To date, several murine models of metaplasia in the stomach have been developed [36]. Here, we briefly review these models and the insights they have provided into the molecular mechanisms and cellular origins of metaplastic lesions (Table 2).

Metaplasia in the stomach in mice was first seen in a model of long-term *Helicobacter felis* infection, which triggers chronic inflammation, atrophy, and mucous-producing metaplasia [37,38,39]. The development of such histological changes in the proximal stomach was accelerated in insulin-gastrin (INS-GAS) mice, which exhibit higher levels of serum amidated gastrin [38,40]. Interestingly, gastrin protects against *Helicobacter*-induced carcinogenesis in the distal stomach antrum, suggesting that gastrin has different functions in the corpus versus antrum [41,42,43]. The anti-tumorigenic effect of gastrin is thought to be caused in part by suppression of the epigenetic silencing of *Tff1*, which encodes a tumor suppressor that attenuates nuclear factor kappa B (NF–κB) signaling [44,45]. Certain *H. pylori* strains, particularly the PMSS1 strain, which has a functional *cag* pathogenicity island (PAI), can also trigger atrophic gastritis and metaplasia [46,47,48]. In these models of *Helicobacter* infection, the development of host immune and inflammatory responses is the key to the induction of atrophic and metaplastic changes. For example, infection of T-cell-deficient mice with *H. felis* created no detectable gastric pathology despite high levels of colonization [39]. However, in Myd88-deficient mice, in which immunological tolerance is impaired, the gastric pathology was accelerated in response to *H. felis* [49,50]. Obesity, which causes low-grade systemic inflammation, exacerbated the gastric pathology induced by *H. felis* by enhancing the Th17 response [51]. Mice infected with PMSS1 during the neonatal period were protected from gastritis, atrophy, and metaplasia by immunological tolerance [47]. Furthermore, inflammatory responses and metaplastic changes were attenuated under germ-free conditions [52], suggesting a role for commensal gastric microflora in disease progression.

On the other hand, several cytokines appear to be essential in the development of metaplasia. Overexpression of interlukin (IL)-1β in the gastric epithelium caused gastric inflammation, atrophy, metaplasia, and even development of invasive lesions (at later time points) [53], whereas IL-1 receptor-knockout mice were protected from the pathological process induced by *H. pylori* PMSS1 [48]. Transgenic expression of IFN-γ, SDF-1, or IL-8 in mice accelerated gastric inflammation and metaplasia during *Helicobacter* infection, via different mechanisms [54,55,56]. IL-33 triggered a Th2 response and metaplastic changes in the stomach when administered systemically [57].

However, the metaplastic lesions in these mouse models do not express Cdx family proteins and do not contain goblet cells or other intestine-specific cell types. Thus, metaplasia in most mouse models appears to differ from IM in humans. Rather, the mouse lesions express trefoil factor 2 (TFF2, also known as spasmolytic polypeptide), and the lesion is thus termed spasmolytic polypeptide-expressing metaplasia (SPEM) [37]. SPEM expresses other neck cell markers such as MUC6 and GSII and shows strong positive Alcian blue staining because of abundant mucins in the cytoplasm. Histologically, SPEM of the *Helicobacter* infection models can be divided into two subtypes: mucous metaplasia and pseudopyloric metaplasia [58]. Although these two lesions are morphologically distinct, both express similar neck cell markers and contain mucin-rich cells that exhibit Alcian blue positivity; both can thus be termed SPEM. However, given the expression of TFF2 and other markers in normal neck cells, any diagnosis of SPEM needs to be performed quite carefully. Alcian blue staining must be combined with analysis of recently discovered SPEM-specific markers such as CD44 variants and Sox9 when diagnosing genuine metaplasia [48,59].

Similar SPEM lesions have been reported in inflamed human stomachs by immunohistochemical analysis of TFF2 [60,61] (Table 1). It should be noted that SPEM glands in both human and mouse stomachs are similar in many aspects, but they need to be sharply distinguished from IM since their morphology and expressed markers are quite different. Analyses of resected human gastric corpus specimens revealed regions containing compound glands in which SPEM cells were observed in the deeper regions of the glands and IM lineages in the luminal portions of the glands, suggesting that IM may develop from the more proliferative SPEM [61], or that IM and SPEM share a common origin in such glands. In any case, whether SPEM in the human stomach has any relevance to gastric cancer remains unclear. Interestingly, *Tff2*-knockout mice were particularly susceptible to gastric inflammation and cancer, and TFF2 expression decreased during gastric carcinogenesis because of methylation of the *TFF2* gene promoter, suggesting that TFF2 or SPEM may act as a tumor suppressor [58,62].

No mouse model has yet been reported to exhibit complete IM with goblet cells, except for transgenic mouse lines expressing Cdx1 or Cdx2 in the gastric epithelium [63,64,65]. Interestingly, Cdx2 transgenic mice develop gastric adenocarcinoma at 100 weeks of age, suggesting that aberrant Cdx2 expression may induce malignant transformation in the stomach [66]. In contrast, several mouse models with epidermal growth factor receptor (EGFR)–Ras–mitogen activated protein kinase (MAPK) pathway activation in the gastric epithelium exhibited rapid development of SPEM-like metaplasia. Mutant *Kras* transgenic mouse lines have been widely used; mutant *Kras* is driven by various constitutive or inducible promoters such as K19 (constitutive), *K19*–CreERT, *Ubc9*–CreERT, *Mist1*–CreERT, *Tff1*–CreERT, eR1–CreERT, and *Lgr5*–CreERT [67,68,69,70,71,72,73,74]. These mice display rapid atrophy development, foveolar hyperplasia, and SPEM exhibiting positive Alcian blue staining and positive TFF2, GSII lectin, and CD44v9, but mostly negative Cdx1/2 immunostaining. Mutant Braf expression under the control of an inducible *Tff1* promoter and overexpression of the EGFR ligand TGFα also triggered similar phenotypes [72,75,76]. Interestingly, mice deficient in amphiregulin, another EGFR ligand, developed SPEM and dysplasia at later time points [77], although the mechanisms of this seemingly counterintuitive result are not fully understood. Inhibition of the MAPK pathway prevented and reversed *Kras*-induced metaplasia, suggesting the potential utility of such inhibitors for treatment of metaplasia [71]. The bone morphogenetic protein (BMP) pathway [78,79] may play a role in metaplasia development via Smad phosphorylation, perhaps acting cooperatively with MAPK activation [80,81]. 

Other groups have developed short-term drug-induced metaplasia models using DMP-777, L-635, and high-dose tamoxifen [82,83,84,85]. The metaplastic lesions of these models express the neck cell marker TFF2 and low level of murine chief cell markers (e.g., intrinsic factor), but the expression of these markers is rapidly reversible after discontinuation of drug treatment. Acute recruitment of macrophages and production of inflammatory cytokines may play roles in the observed responses [86,87]. Although these models are useful for exploring the processes of acute injury and regeneration because of the relatively short time courses of the experiments compared with those performed in other transgenic or infection models, the reversible nature of the lesions contradicts the notion that metaplasia is caused by irreversible reprogramming of cell differentiation. Indeed, the short-term metaplastic lesions induced by high-dose tamoxifen do not exhibit markers of long-term SPEM, such as Alcian blue staining and CD44v9/Sox9 positivity, although recent reports have shown that CD44v9 expression was somewhat elevated after DMP-777 or L-635 treatment [87,88]. Parietal cell loss was initially believed to be the main trigger of metaplastic development in these models, but this has been questioned by a recent study showing that parietal cell loss alone did not induce metaplasia [85]. In our opinion, the mechanisms of drug-induced SPEM are essentially regenerative, thus reflecting responses to acute injury, leading to hyperplasia of neck/chief cell lineages.

Recently, Menheniott et al. reported that mice deficient in gastrokine-2, a secreted protein normally expressed by gastric surface mucous cells, spontaneously developed Alcian blue-positive mucous metaplasia and mucous neck cell hyperplasia and exhibited an exacerbated pathology when infected with *H. pylori* [89]. Together with the anti-tumorigenic effect of TFF1, which is also secreted by surface mucous cells, these results suggest that such cells play protective roles against gastric disease [45].

## 3. Origins of Metaplasia

As mentioned above, the field cancerization phenomenon suggests that both human IM and dysplasia are of stem cell origin [32]. Immunohistochemical analyses of SPEM/IM compound glands in the human stomach revealed that Ki67-positive proliferating cells are located mainly in the IM component which constitutes the upper half of the gland, suggesting that IM originates from the stem cell zone at the isthmus, not at the bottom, of the gland [61].

In mouse models, the origin of metaplasia in the stomach has been much debated. Given the abundant proliferation evident in the isthmus of the stem cell zone, metaplasia is thought to originate from isthmal stem or progenitor cells [59,90,91]. However, several groups have recently proposed a model whereby metaplasia originates from mature gastric chief cells through transdifferentiation [92,93]. Lineage tracing experiments using CreERT-expressing lines crossed to *Rosa26* reporter lines were used to analyze the origin of metaplasia, but the results were quite complex. Initially, *Mist1*–CreERT mice were used as a chief cell-specific line; metaplasia in these mice develops from *Mist1*+ cells [71]. However, later, our group revealed that not only chief cells but also isthmal stem cells express *Mist1*-CreERT [70]. Another group reported metaplasia development in eR1–CreERT mice crossed to mutant *Kras* mice, but both chief cells and isthmus stem cell/progenitor cells broadly expressed eR1–CreERT [73]. *Lgr5* may be expressed more specifically in basal chief cells, and it has been reported that *Lgr5*+ chief cells do not give rise to metaplasia in *Lgr5*–CreERT–IRES–EGFP mice [94]. In addition, *Mist1*–CreERT crossed to LSL–*Kras*^G12D^ mice developed metaplasia even after *Lgr5*+ cell ablation in *Lgr5*–DTR mice [70]. Although these data strongly suggest that *Lgr5*+ chief cells are not the precursor to metaplasia, a group in Singapore recently reported that *Lgr5*+ chief cells can give rise to metaplasia after acute injury in a separate *Lgr5*–CreERT mouse line [74]. 

Several factors may influence the results and conclusions drawn in lineage tracing experiments. Tamoxifen used to activate the CreERT recombination can itself trigger acute injury of the gastric epithelium [84]. Genes of interest are often haploinsufficient after insertion of CreERT sequences that may affect cellular kinetics [70,95]. The frequency and efficiency of recombination varies depending on the *Rosa26* reporter lines used in various experiments [96]. More specific Cre drivers or Cre-independent recombination models might be useful to avoid such influences, but we emphasize at this point that detailed careful observation is critical to obtain more accurate interpretations and conclusions. Analysis at various time points (particularly earlier time points), using different doses of tamoxifen, and examination of multiple sections or reconstructed 3D images, is essential to determine the exact cell fates in various models. 

## 4. Cellular Responses during *H. pylori* Infection

Many studies on bacterial virulence factors and the host cellular responses to *H. pylori* infection have appeared [97,98,99,100,101,102]. Interactions between virulence factors and epithelial cells deregulate or activate various signaling pathways such as those involving NF–κB and Ras–MAPK kinases. Many studies on the cellular responses to *H. pylori* infection were based on in vitro experiments using gastric cell lines. The effects of signaling pathway dysregulation in gastric epithelial cells have also been investigated in vivo, using mouse models such as those discussed above. Recently, a gastric organoid system has been used to investigate the interactions between *H. pylori* and epithelial cells [103].

One of the most important and best-studied virulence factors is the cytotoxin-associated gene A (CagA) and the associated type IV secretion system (T4SS), which are associated with various gastric pathologies including gastritis, gastric ulcers, and cancer [104,105]. These genes are located within the *cag* PAI, a 40-kb DNA segment containing a cluster of 30 genes [106]. CagA is delivered to the gastric epithelial cell cytoplasm via T4SS and then undergoes phosphorylation of the EPIYA motifs by host Src-family kinases [107,108,109,110]. Phosphorylated CagA binds to and activates the SH2-domain-containing protein tyrosine phosphatase (SHP2) [111]. CagA-activated SHP2, in turn, activates the Ras–MAPK pathway, which is involved in cell proliferation and perhaps the development of metaplasia [112,113]. Transgenic mouse models have been used to show that systemic expression of CagA triggers spontaneous development of gastric tumors, and that the tumorigenic effect of CagA is dependent on EPIYA motif phosphorylation [114]. EGFR, an upstream of the Ras–MAPK pathway, is also activated by *H. pylori* [115]. Basu et al. reported that the *H. pylori* secreted protein HP0175 activates EGFR via interaction with TLR4. The importance of EGFR activation in vivo was shown in mouse and Mongolian gerbil models [116]. These studies revealed the potential therapeutic implications of EGFR inhibition in patients with *H. pylori* gastritis.

CagA exerts a wide range of effects on gastric epithelial cells. One example is Wnt/β-catenin pathway activation, which is particularly important for the development of colorectal neoplasia [117]. CagA activates the Wnt/β-catenin pathway via interaction with E-cadherin, independent of tyrosine phosphorylation status. Wnt signaling can affect the expression of Cdx1, which was reported to be a Wnt target gene, and may be associated with IM development in the stomach [118]. In mouse models, however, Wnt signaling activation alone was not sufficient to induce Cdx expression or metaplastic changes in the corpus epithelium, as discussed above [70]. CagA was also involved in activation of the signal transducer and activator of the transcription 3 (STAT3) pathway [119,120]. The IL-6/STAT3 pathway was shown to play a role in gastric carcinogenesis in mouse models [121]. Other effects of CagA on gastric epithelial cells include cytoskeletal reorganization, increased proliferation and mobility, junctional defects, and polarity loss [122,123,124]. However, it remains unclear whether CagA is indispensable for gastric pathology, or whether other components of the *cag* PAI play more important roles. Importantly, *H. pylori* with the *cag* PAI also activates non-classical, stress-induced MAPK pathways, including those involving the JNK and p38 MAPKs. Upstream molecules of these pathways (including MAP3Ks such as transforming growth factor beta-activated kinase 1 [TAK1] and apoptosis signal-regulating kinase 1 [ASK1]) have been identified [121,125]. In mouse models, knockout of c-Jun N-terminal kinase 1 (JNK1) or ASK1 suppressed gastric tumorigenesis, likely via regulation of apoptotic responses and cyclin D1/E2F1-dependent cell cycle signaling [126,127,128]. *H. pylori-*derived lipopolysaccharide is another virulence factor involved in the activation of stress-induced MAPK pathways and subsequent apoptosis [129].

The NF–κB pathway, which regulates the expression of a wide variety of inflammatory cytokines, is another critical pathway involved in *Helicobacter*-induced gastritis and metaplasia [130,131]. Many in vitro studies have shown that CagA and/or the *cag* PAI activates the NF–κB pathway, triggering IL-8 or IL-32 upregulation and anti-apoptotic responses [132,133,134,135]. Interestingly, *H. pylori*-induced IL-8 upregulation is in part mediated by RhoA [136], which was recently reported to be frequently mutated in a subset of human gastric cancers [137,138,139,140]. Various mechanisms are involved in *cag* PAI-dependent NF–κB activation [125,141,142,143]. The toll like receptor (TLR) pathways, which are stimulated by bacterial lipopolysaccharide, trigger NF–κB activation in either a *cag* PAI-dependent or -independent manner [144,145]. Bacterial peptidoglycan can be delivered to gastric epithelial cells via T4SS, where it activates NF–κB via Nod1 [146]. Although it has been reported that NF–κB signaling in gastric epithelial cells may protect against *H. felis*-induced gastritis and the subsequent development of dysplasia [147], our group has suggested that NF–κB activation in gastric epithelial cells promotes gastric carcinogenesis via IL-1α production in a chemically induced mouse tumor model [148]. Chronic inflammation, mediated principally by NF–κB signaling, promoted DNA methylation in gastric epithelial cells via activation of DNA methyltransferase 1 [149,150]. Although aberrant DNA methylation may be involved in the reprogramming of epithelial differentiation and metaplastic changes, no clear evidence supporting this idea has yet been presented. 

## 5. Conclusions

As discussed above, recent lineage tracing experiments in mouse models and clonality analyses of human samples together suggest that tissue-resident stem cells likely give rise to both metaplasia and cancer, although other possibilities (such as cancer originating from bone-marrow-derived cells) have been suggested [151]. Ras–MAPK pathway activation, which is a direct effect of *H. pylori* infection (particularly by *cag* PAI-positive strains) of gastric epithelial cells, appears to be key in terms of long-term metaplasia, similar to what is seen in mouse models of *Helicobacter* infection and mouse models of transgenic activation of *Kras*.

However, IM in humans with gastritis is quite stable and almost irreversible, even after *H. pylori* eradication and subsidence of inflammation. One explanation for this stability is genetic and/or epigenetic reprogramming in stem cells, which may result in generation and maintenance of metaplastic glands.

An alternative hypothesis is that niche factors supporting tissue-resident stem cells and regulating epithelial differentiation are altered after long-term inflammation to activate MAPK pathways in stem cells or deregulate epithelial differentiation. Such niche factors potentially include immune cells, vascular endothelial cells [70], nerves [152,153,154], and fibroblasts, some of which may be derived from bone marrow [155]. Studies on such factors are needed to gain a deeper understanding of gastric pathophysiology and to develop useful clinical treatments in the future. 

## Figures and Tables

**Table 1:** Characteristics of metaplastic lesions in the human stomach.

Type of Metaplasia	Histological Characteristics	Representative Markers
Intestinal metaplasia (IM)	Intestine-like gland structures Intestine-specific cell lineages	CDX1, CDX2 MUC2, TFF3, Villin Alcian blue staining at pH 2.5
Complete IM	Small intestinal phenotype Enterocytes with brush borders Well-defined goblet cells Paneth cells	Loss of gastric mucins (MUC1, 5AC, 6)
Incomplete IM	Colonic gland-like structures Irregular mucin droplets	Brown staining by high iron diamine (HID) Both gastric and intestinal mucins may be present
Spasmolytic polypeptide-expressing metaplasia (SPEM)	Deep antral gland-like structure Expansion of a mucous neck cell-like lineage	TFF2, MUC6, GSII Alcian blue staining at pH 2.5

CDX1: caudal type homeobox 1; CDX2: caudal type homeobox 2; GSII: Griffonia simplicifolia lectin II; TFF2: trefoil factor 2; TFF3: trefoil factor 3.

**Table 2:** Characteristics of mouse models of stomach metaplasia.

Type of Metaplasia	Histological Characteristics	Representative Markers
SPEM in *Helicobacter* infection or after transgenic activation of Ras–MAPK signaling	Accompanied by atrophy, foveolar hyperplasia, and inflammation Dysplasia at later time points No development of IM Generally irreversible	TFF2, MUC6, GSII CD44v9, SOX9 Alcian blue staining at pH 2.5
IM in transgenic Cdx1/2 mouse models	Complete IM Enterocytes with brush borders Well-defined goblet cells Paneth cells Irreversible	CDX1 or CDX2 MUC2, TFF3 Alcian blue staining at pH 2.5 Brown staining by HID
Acute drug-induced injury (DMP-777, L-635, high-dose tamoxifen)	Acute loss of parietal cells and mature chief cells with inflammatory infiltration Reversible after discontinuation of drug treatment	TFF2, MUC6, GSII Low expression of gastric intrinsic factor CD44v9 (DMP-777 and L-635)

CD44v9: CD44 variant 9; CDX1: caudal type homeobox 1; CDX2: caudal type homeobox 2; GSII: Griffonia simplicifolia lectin II; SOX9: sex determining region Y-box 9; TFF2: trefoil factor 2; TFF3: trefoil factor 3.
